# Selective secretion of microRNAs from lung cancer cells via extracellular vesicles promotes CAMK1D-mediated tube formation in endothelial cells

**DOI:** 10.18632/oncotarget.19996

**Published:** 2017-08-07

**Authors:** James Lawson, Christopher Dickman, Sara MacLellan, Rebecca Towle, James Jabalee, Stephen Lam, Cathie Garnis

**Affiliations:** ^1^ Department of Integrative Oncology, British Columbia Cancer Research Centre, Vancouver, BC, Canada; ^2^ Division of Otolaryngology, Department of Surgery, University of British Columbia, Vancouver, BC, Canada

**Keywords:** MiRNA, serum miRNA, lung adenocarcinoma, extracellular vesicles

## Abstract

Extracellular vesicles (EVs) are key signaling mediators between cancer cells and their supporting stroma, and regulate critical processes such as invasion, metastases, and angiogenesis. We have identified a subset of miRNAs (miR-142-3p, miR-143-3p, miR-145-5p, miR-150-5p, miR-223-3p, miR-451a, miR-486-5p, miR-605-5p) that are enriched in lung adenocarcinoma extracellular vesicles compared to the donor cells from which they were derived. Two well-known tumor suppressors, miR-143-3p and miR-145-5p, were also enriched in serum samples collected during surgery from blood vessels draining directly from lung adenocarcinoma tumor beds. Recently, both miRNAs were found to promote neoangiogenesis in endothelial cells in mouse models of lung adenocarcinoma through targeting of CAMK1D, an inhibitory kinase that can impair angiogenesis when over-expressed. We show that the transfer of miR-143-3p and miR-145-5p within extracellular vesicles from lung adenocarcinoma cells to endothelial cells reduces the levels of CAMK1D and increases tube formation by endothelial cells. This finding suggests that transfer of miRNAs within extracellular vesicles is a method of communication between cancer and endothelial cells which promotes angiogenesis while simultaneously removing tumor suppressive miRNAs within the tumor cells, thus driving tumorigenesis.

## INTRODUCTION

Lung cancer remains the leading cause of cancer death worldwide, and lung adenocarcinoma (LAC), the most common histological subtype, has a dismal 5-year survival rate of ~16% [1, 2]. While advances have been made in treatments, survival rates remain poor [3]. With a greater understanding of the molecular mechanisms driving LAC, development of innovative treatment options to better combat this disease may become possible.

Recently, there has been increased interest in the role of extracellular vesicles (EVs) in tumorigenesis. EVs are a heterogeneous group of secreted small membrane bound vesicles. They are known to play a variety of roles in cancers, including establishing a pre-metastatic niche, conferring drug resistance, and promoting angiogenesis through cell-cell communication [4, 5]. EVs contain a variety of cargo that determine EV function, such as mRNAs, miRNAs, and proteins [6]. MiRNAs are small non-coding RNAs that post-transcriptionally regulate expression of protein coding genes [7, 8]. MiRNAs are known to be involved in numerous biological processes, in disease development, and are highly deregulated in cancer [9]. In breast cancer, EVs containing miR-181c are able to promote metastases to the brain through destruction of the blood brain barrier [10]. Other roles for EV miRNA include promoting chemotherapy resistance. For example, in breast cancer, the transfer of EVs containing miR-100, miR-122 and miR-30a from resistance MCF-7 breast cancer cells are known to promote chemotherapy resistance to MCF-7 drugs sensitive cells [11]. Finally, the role of tumor derived EVs in promoting angiogenesis has been well established. Liu et al. found that EVs containing miR-21 are able to increase VEGF levels in recipient cells through targeting of STAT3 [12–14].

The loading of miRNAs into EVs is not a passive process and can differ depending on cell type and disease state [9]. Certain miRNAs are selected for EV loading and exclusion from cells, while others are selectively retained by cells, suggesting a biological role for these miRNAs in cancer [15]. Sorting of miRNAs into EVs has been previously reported through hnRNPA2B1, an RNA binding protein regulated by sumoylation and able to bind the RNA motif GGAG, however it does not fully explain all miRNA sorting into EVs [16]. Herein, we report on miRNAs that are selectively enriched within EVs of LAC cells and show that EVs enriched for specific miRNAs enter endothelial cells and promote blood vessel formation by altering the activity of CAMK1D, an anti-angiogenic kinase.

## RESULTS

### Identification of miRNAs enriched in the EVs of LAC cells

**Figure 1:**
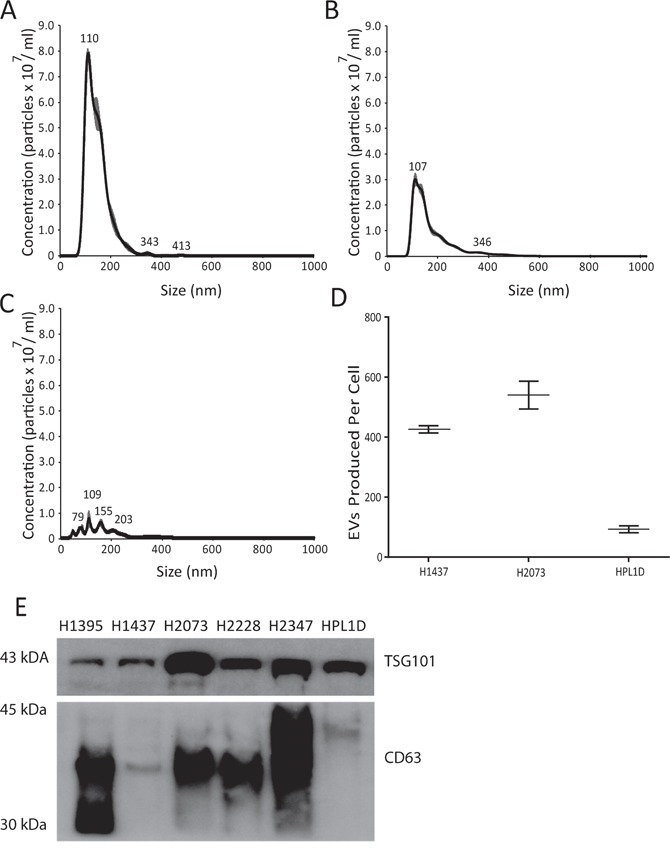
Extracellular vesicle identification **(A-C)** Nanoparticle Tracking Analysis of extracellular vesicles, quantifying size and concentration using NanoSight: (A) H1437 cell line (B) H2073 cell lines (C) HPL1D cell line. **(D)** Comparison of number of EVs produced per cell over 48 hours for H1437, H2073 and HPL1D cell lines. **(E)** Western blot confirmation of EV associated proteins CD63 and TSG101.

EVs from LAC cell lines H1395, H1437, H2073, H2228, and H2347 were collected by differential ultracentrifugation [17]. Analysis by nanoparticle tracking analysis (NTA) revealed the majority of EVs were ~110 nm in size (Figure 1A and 1B). This size range is consistent with the size of exosomes, a subset of EVs. Furthermore, EV presence was confirmed by western blot for common EV markers CD63 and TSG101 (Figure 1E). RNA from both donor cells and isolated EVs was extracted using the miRCURY™ RNA Isolation (Exiqon) kit and profiled for miRNA expression using microRNA Ready-to-Use PCR, Human panel I+II, V4.M (Exiqon).

Of the 742 miRNAs examined, an average of 264 miRNAs were detected in the EV fractions and 258 miRNAs in the donor cell fractions. For each donor cell-EV pair on average, four miRNAs were uniquely expressed in the donor cell fraction and 12 miRNAs were uniquely expressed in the EV fraction. We used a 4-fold expression cut-off between the EV and the donor cell miRNA profiles to identify miRNAs that were selectively enriched in EVs. On average 13 miRNAs were selectively enriched in EVs across all cell lines. Nine miRNAs were recurrently enriched in the EVs of ≥3 of the five cell lines profiled (Table 1). On average, 14 miRNAs were selectively retained in each donor cell line as compared with associated EVs. However, none of these donor cell-enriched miRNAs were commonly enriched across the entire panel of cell lines. Recurrent miRNAs were validated in the matched donor cell and EV RNA samples using individual qRT-PCR reactions. A 4-fold difference was observed for all candidate miRNAs, except for miR-144-3p, which was eliminated from further analysis.

**Table 1:** Profiling fold change of miRNAs enriched by 4-fold in the EVs of at least 3 LAC cell lines using microRNA qRT-PCR

miRNAs	Number of cells lines with miRNA enriched in EVs >4-fold (n=5)	H1395 fold change	H1437 fold change	H2073 fold change	H2228 fold change	H2347 fold change
miR-451a	5	273.22^*^	214.29^*^	50.42^*^	458.11^*^	83.26^*^
miR-142-3p	5	30.93	71.08^*^	17.70^*^	95.84^*^	18.68
miR-223-3p	5	179.01^*^	41.20^*^	12.10^*^	55.89^*^	20.46^*^
miR-144-3p	5	51.05^*^	32.55^*^	6.38^*^	59.16^*^	8.49^*^
miR-605-5p	4	0.92	17.11^*^	5.20^*^	54.51^*^	8.90^*^
miR-150-5p	4	79.45^*^	5.35	2.93^*^	21.15^*^	8.73^*^
miR-145-5p	4	31.25^*^	9.19^*^	7.39^*^	3.10^*^	4.15
miR-486-5p	3	38.42	9.62^*^	3.07^*^	6.88^*^	3.52^*^
miR-143-3p	3	20.07^*^	13.13^*^	1.76^*^	11.24^*^	1.23

^*^: Indicates fold change where no miRNA was detected intracellularly and intracellular Ct levels was set to threshold cut off of 35 Cts for fold change analysis.

To determine whether the EV-enriched miRNAs were tumor cell specific, we isolated EVs from a normal epithelial lung cell line (HPL1D). RNA was extracted from donor cells and EVs were isolated as above. Using qRT-PCR, we determined the expression of our eight candidate miRNAs in the two RNA isolates. We found four of our eight miRNA candidates were also enriched within the EVs of HPL1Ds (miR-142-3p, miR-150-5p, miR-223-3p, and miR-451a); however, the extent of the enrichment observed in the tumor EVs was at least double that observed within the HPL1D EVs (Supplementary Figure 1). This difference cannot be attributed to differences in miRNA expression in donor cells, as cellular expression of these miRNAs is similar between HPL1Ds and the tumor lines. Additionally, NTA analysis revealed that EV production from HPL1D cells was less than 25% of what was observed in LAC cells: while HPL1D cells produced ~92 EVs per cell over 48 hours, H1437 and H2073 cells produced ~425 and ~540 EVs per cell over 48 hours, respectively (Figure 1). In addition, while some miRNAs are commonly selected for EV export between tumor and normal cells, the enrichment in the tumor EVs is far greater (Supplementary Figure 1) and EV production from the tumor cells is on average ten times higher than normal lung cell production (Supplementary Figure 2).

### Inhibition of EV release increases the levels of candidate miRNAs within LAC cells

**Figure 2:**
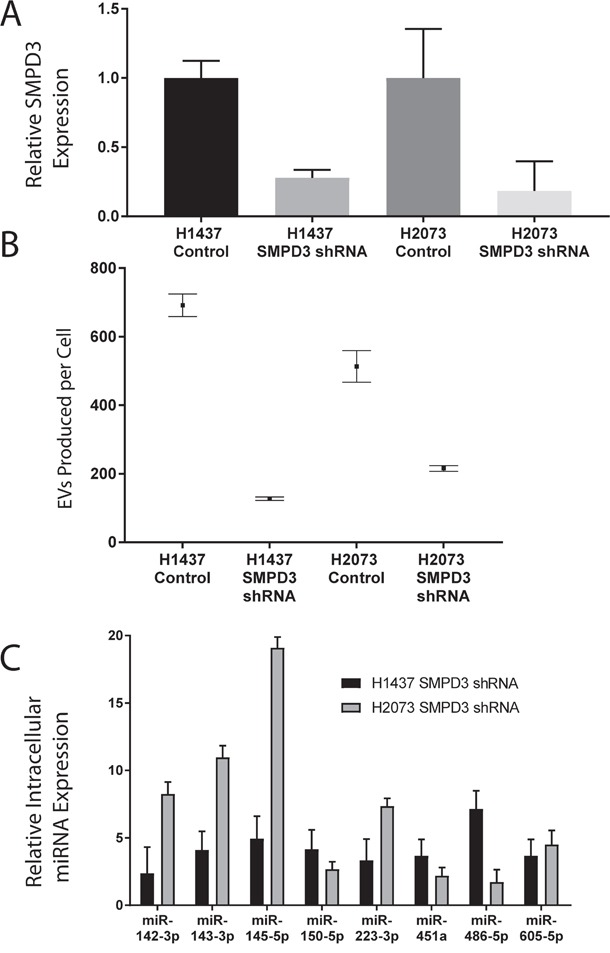
Analysis of EV inhibition using SMPD3 **(A)** Cellular inhibition of SMPD3 in H1437 and H2073 cell lines using shRNAs against SMPD3 confirmed using qRT-PCR. **(B)** EV secretion analysis measuring EVs produced per cell over 48 hours of H1437 and H2073 cells lines using SMPD3 shRNA inhibition using Nanosight. **(C)** Fold change analysis of candidate miRNAs from H1437 and H2073 intracellular RNA with SMPD3 shRNA inhibition.

To confirm that the detected candidate miRNAs were isolated from EVs, as opposed to co-precipitating factors, we inhibited the release of EVs by silencing SMPD3, a critical protein in ceramide-dependent exosome biogenesis [18]. We would expect the miRNA cargo within the EVs to remain in the cell and therefore show increased cellular expression. SMPD3 was inhibited using shRNAs and a ~75% knockdown was observed in both H2073 and H1437 cell lines (Figure 2A). When SMPD3 was inhibited in H1437 cells EV production decreased 83%, in H2073 cells we observed a ~60% decrease in EV production (Figure 2B) and a cellular increase in all candidate miRNAs was noted in both cell lines (Figure 2C).

### MiR-143-3p and miR-145-5p are enriched within serum draining directly from LAC tumors

We next undertook qRT-PCR profiling of miRNAs in serum samples obtained from the pulmonary venous effluent draining directly from the tumor vascular bed. These serum samples were collected during surgical resection of LAC, as were matched samples from peripheral blood vessels (Supplementary Table 1). MiRNAs known to be influenced by hemolysis were removed from analysis [19]. On average, 169 miRNAs were detected in each sample, 52 miRNAs were expressed in all samples, 72 miRNAs were enriched in each tumor effluent sample by at least 4-fold, and only 12 miRNAs were enriched in peripheral samples by at least 4-fold. Twenty-two miRNAs were consistently enriched by at least 4-fold in the tumor effluent samples in at least half of the ten paired samples, while no miRNAs were recurrently down-regulated in tumor effluent samples (Supplementary Table 2). Five of our eight EV miRNA candidates (miR-142-3p, miR-150-5p, miR-223-3p, miR-451a, miR-486-5p) were shown to be increased by hemolysis and were thus excluded from analysis. Of the remaining EV miRNA candidates, miR-143-3p and miR-145-5p were enriched in tumor draining effluent samples. These miRNAs both exhibited effluent sample enrichment in six of ten cases and exhibited 9.1-fold and 13.8-fold average over-expression in tumor effluent samples as compared to peripheral samples. Given their increased EV expression in both LAC cell lines and LAC patient serum samples, we reasoned that EV-mediated miR-145-5p and miR-143-3p activity might promote lung tumorigenesis.

### MiR-145-5p and miR-143-3p promote tube formation in endothelial cells

Recent work demonstrated that miR-145-5p and miR-143-3p are capable of promoting neoangiogenesis by targeting CAMK1D in endothelial cells, and that loss of the miR-145/miR-143 cluster in these cells led to decreases in neoangiogenesis via CAMK1D [20]. It was suggested that miR-143/145 was not expressed by the tumor epithelial cells, but rather was produced by a subset of endothelial cells driving neoangiogenesis. We show that miR-145-5p and miR-143-3p are in fact produced by lung adenocarcinoma cells; however, no expression is observed in the epithelial cells, as these miRNAs are efficiently packaged into EVs and exported from the cell.

**Figure 3:**
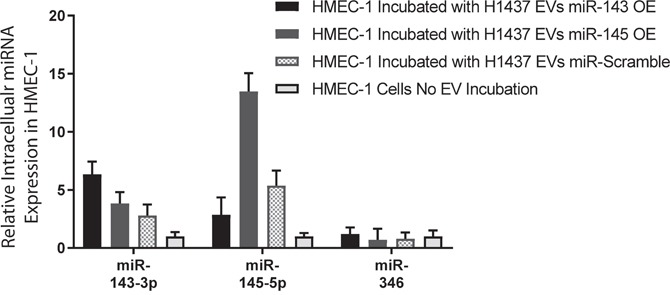
Lung adenocarcinoma EV miRNAs are taken up by HMEC-1 cells Intracellular fold change analysis of miR-143-3p, miR-145-5p and miR-346 in HMEC-1 cells incubated with EVs from H1437 cells over expressing miR-143, miR-145 and miR-Scramble, compared to HMEC-1 cells receiving no EV incubation.

To interrogate the impact of EV-associated miR-143-3p and miR-145-5p, the levels of these miRNAs within EVs must be manipulated. We over-expressed miR-143, miR-145 and a miR-Scramble individually in H1437 cells, and H2073 cells which resulted in >100-fold over-expression of these miRNAs in the EVs derived from these cells. EVs over-expressing miR-143 and miR-145, as well as miR-Scramble EVs (using a miRNA scramble, but still expressing normal levels of miR-143-3p and miR-145-5p in the EVs) were collected and incubated with HMEC-1 cells for 24 hours, along with HMEC-1 cells receiving no EVs. Following this, EVs were removed, the cells were washed and cellular RNA from HMEC-1 cells was extracted. MiR-145-5p and miR-143-3p increased by ~14-fold and ~6-fold in HMEC-1 cells incubated with EVs over-expressing miR-143 and miR-145 respectively when compared to HMEC-1 cells that were not incubated with EVs (Figure 3). HMEC-1 cells incubated with miR-Scramble over-expressing EVs (with normal miR-145-5p and miR-143-3p levels within EVs) showed an increase in miR-145-5p >5 fold and a slight increase in mR-143-3p by >2 fold (Figure 3). On the other hand, HMEC-1 cells showed no significant changes in intracellular levels of a miRNA (miR-346) that was not found in EVs from H1437 cells (Figure 3).

**Figure 4:**
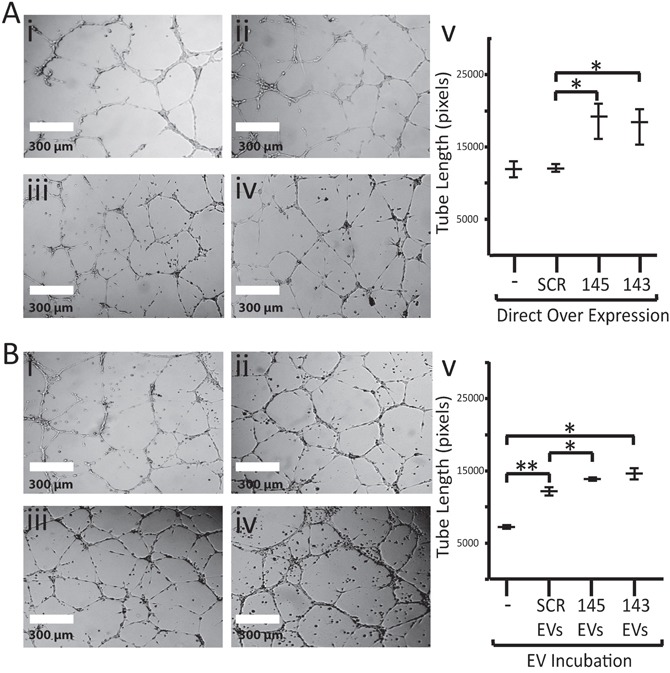
Impact of miR-143 and miR-145 on tube formation **(A)** Tube formation photos of HMEC-1 cells at 10x magnification with i) no vector ii) control vector iii) miR-143-3p over-expression vector or iv) miR-145-5p over-expression vector v) graph of tube formation on HMEC-1 endothelial cells with no direct over expression (-) and direct over expression of miR-scramble (SCR), miR-143 (143) and miR-145 (145). **(B)** Tube formation photos of HMEC-1 cells at 10x magnification incubated with EVs from H1437 cells i) No EVs ii) miR-Control over-expressing EVs iii) miR-143-3p over-expressing EVs or iv) miR-145-5p over-expressing EVs v) graph on tube formation for HMEC-1 cells incubated with no EVs (-), H1437 miR-Scramble overexpressing EVs (SCR EVs), H1437 miR-143 overexpressing EVs (143) and H1437 miR-145 overexpressing EVs (145) ^*^ = p-value < 0.05 ^**^ = p-value < 0.01.

We next sought to determine if the transfer of EVs, and in particular miR-143-3p and miR-145-5p, within the EVs is capable of inducing tube formation changes in endothelial cells. HMEC-1 cells were grown without EVs or in the presence of EVs over-expressing miR-143, EVs over-expressing miR-145 or control EVs (endogenous EV levels of miR-143 and miR-145). All HMEC-1 cells exposed to EV incubation treatments formed tubes after 16 hours (Figure 4E–4H). The addition of EVs from H1437 miR-Scramble over-expressing cells (with normal miR-145-5p and miR-143-3p levels within EVs) significantly increased the ability of HMEC-1 cells to form tubes compared to the HMEC-1 cells receiving no EVs (p<0.01) (Figure 4I). The addition of EVs over-expressing miR-145-5p or miR-143-3p further increased the length of tubes formed compared to miR-Scramble over-expressing EVs (Figure 4I). Significance was reached for miR-145 (p=0.03) but not for miR-143 (p=0.1) (Figure 4I).

To determine if the EV-transferred miRNAs target CAMK1D in HMEC-1 cells, protein lysates from HMEC-1 cells were collected after incubation with EVs or with the negative control (incubation with no EVs). All HMEC-1 cells that received EVs containing miR-143-3p or miR-145-5p showed a decrease in CAMK1D protein by western blot. The addition of miR-Scramble over-expressing EVs from H1437 cell lines caused a decrease in the cellular quantity of CAMK1D by 53% compared to the negative control (Figure 3B). A greater decrease was observed in HMEC-1 cells incubated with EVs over-expressing miR-145 or miR-143, showing a 79% and 83% decrease respectively compared to HMEC-1 cells receiving no EVs (Figure 3B).

**Figure 5:**
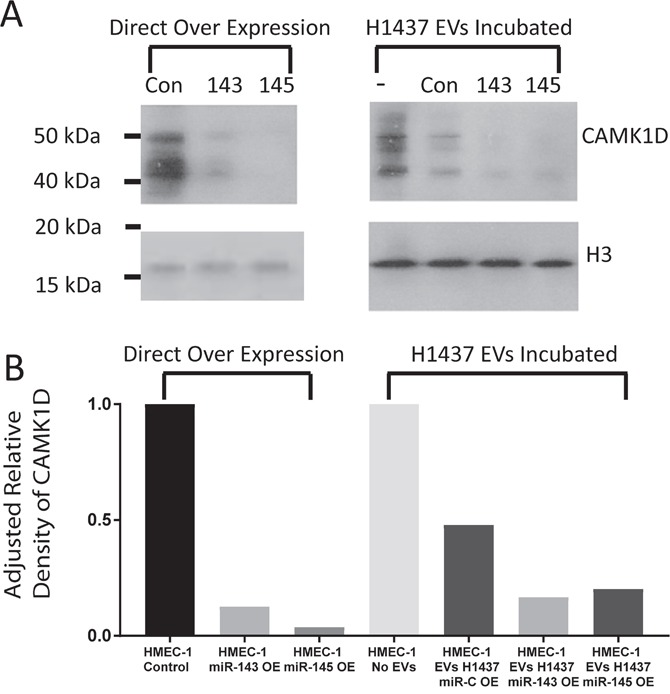
Analysis of CAMK1D targeting by miR-143 and miR-145 **(A)** Western blot of CAMK1D and Histone 3 in HMEC-1 cells after direct over expression of miR-143, miR-145 and miR-Scramble within HMEC-1 cells and after incubation with EVs isolated from H1437 over expressing miR-143, miR-145, miR-Scramble and unmodified cell. **(B)** Densitometry analysis of CAMK1D western blot in Figure 5A.

To confirm that the observed increases in tube formation were at least in part caused by the presence of miR-145-5p or miR-143-3p, we examined the tube forming ability of HMEC-1 cells that directly over-expressed miR-145 and miR-143 (as opposed to being over-expressed via EVs). Figure 4I demonstrates that direct over-expression was capable of increasing tube formation by 36% and 34% for miR-145 and miR-143, respectively (p<0.04 for both). No changes in tube formation were noted in cells transfected with a miR-Scramble vector or in cells that were not exposed to infection. HMEC-1 cells over-expressing miR-143 and miR-145 had decreased CAMK1D protein levels (87% and 96%, respectively) compared to HMEC-1 cells over-expressing the miR-Scramble (Figure 5A and 5B).

We identified a set of miRNAs that are selectively packaged into EVs and released from LAC cell lines. Cross referencing with miRNA profiles obtained from serum collected from blood directly draining from the tumor suggests a biological role for miR-143-3p and miR-145-5p in lung tumorigenesis. These miRNAs were shown to be produced by LAC cells and packaged into EVs. Once packaged the EVs are capable of shuttling miRNA cargo to endothelial cells where miR-143-3p and miR-145-5p down-regulate CAMK1D, promoting angiogenesis.

## DISCUSSION

In the present study, using LAC cell lines, we identified several miRNAs (miR-142-3p, miR-143-3p, miR-145-5p, miR-150-5p, miR-223-3p, miR-451a, miR-486-5p, and miR-605-5p) that are selectively packaged into EVs and released into the tumor microenvironment. The function of tumor-derived EVs is dictated by EV cargo and it is now appreciated that EV cargo differs between cell types and disease state. Therefore, it is imperative to evaluate the function of EVs in LAC in order to understand their role in this disease.

Profiling of a panel of LAC cell line EVs revealed that eight miRNAs were frequently enriched within EVs and absent within the donor cells that gave rise to these EVs. These results suggest the enriched miRNA candidates are sorted and packaged efficiently into EVs, so much so that their intracellular detection in donor cells is difficult with standard molecular techniques. The mechanisms by which miRNAs are packaged into EVs are not well understood. Previous studies have proposed miRNA uptake into EVs occurs through specific miRNA sequences and hydrophobic modifications such as methylation [15, 16]. These mechanisms may explain some miRNA selection, but they do not fully account for the specific cohort of candidate miRNAs that we observe here. For example, miR-451a (which we observe to be enriched in the EVs) also contains a CL (cellular) motif, which has been suggested to mediate exclusion from EVs [16]. Four of our miRNA candidates (miR-142-3p, miR-150-5p, miR-451a, and miR-486-5p) are found within EVs of many cell types including HEK 293T cells, breast cancer cells, and oral cancer cells [21, 22]. These miRNAs are known tumor suppressors that alter activity of many genes involved in tumorigenesis including TGFβR1, MUC4, ARHGAP, and RAB14 [23–26]. Selection of these miRNAs for export would then promote tumor growth by eliminating intracellular tumor suppressive activity of the miRNAs [23–26].

Our remaining miRNA candidates (miR-143-3p, miR-145-5p, miR-223-3p, and miR-605-5p) appear to be more specific to lung adenocarcinoma. These miRNAs are generally considered tumor suppressive miRNAs in many cancer types. MiR-143-3p has been shown to target KRAS and IGF1R in colorectal cancer [27, 28]. MiR-145-5p is also known to target IGF1R in colorectal cancer, as well as EGFR in LAC [28, 29]. Over-expression of miR-223 in ovarian cancer cell lines reduces proliferation and colony formation, also by targeting of IGF1R [30]. MiR-605-5p has not been widely studied; however, it may function in response to stress given some indication that it can enhance transactivation of p53 by inhibiting MDM2 in lung and breast cancer cell lines [31].

There is limited information on the function of these candidate miRNAs in cells of the tumor microenvironment. Continued research into stromal function of EV miRNAs will allow for a greater understanding of tumor-stromal EV signaling in cancer. To the best of our knowledge, this is the first time miR-143-3p and miR-145-5p are reported as enriched within EVs released directly from LAC cells. MiR-143-3p and miR-145-5p are important tumor suppressors in many cancer types including lung, bladder, colon, and breast [32–35].

Based on these findings, the function of selective packaging and release of certain miRNAs is two-fold. The tumor cells selectively package tumor suppressive miRNAs within EVs to eliminate their function intra-cellularly and, in doing so, end up transporting miRNAs to endothelial cells (and possibly other stromal cells), which creates a more favorable tumor microenvironment.

Herein, we also demonstrated that miR-143-3p and miR-145-5p derived from LAC cell EVs can target the endothelial cell function of CAMK1D, a negative regulator of angiogenesis. Dimitrova et al. (2016) first showed that stromal expression of miR-143/miR-145 promotes neoangiogenesis by targeting CAMK1D [20]. They also reported that expression of miR-143/miR-145 occurs in a small population of endothelial cells, but not lung epithelial cells [20]. Our results show that miR-143-3p and miR-145-5p are in fact expressed by LAC cells; however, they are packaged into EVs so efficiently that expression of these miRNAs in the donor cells is typically not detected. Once in the EVs, miR-143/miR-145 can enter endothelial cells and down-regulate CAMK1D protein levels. The depletion of CAMK1D within these cells results in increased tube formation in the endothelial cells. These two results are compatible and may be happening in combination, which may further promote neoangiogenesis.

It is evident that miRNAs within EVs play a significant role in tumorigenesis. We report that 8 miRNAs are frequently enriched within EVs released from LAC cells. MiR-143-3p and miR-145-5p, two miRNAs enriched within LAC EVs, were also found enriched within serum obtained from blood draining directly from LAC tumor bearing lungs indicating a biological significance. Both miRNAs have been previously implicated as mediators of neo-angiogenesis and our analysis shows that when transferred through tumor derived EVs miR-143-3p and miR-145-5p promote tube formation through targeting of CAMK1D in endothelial cells. Our work highlights how certain miRNAs considered to be down-regulated (or absent) in LAC are efficiently selectively packaged and released from the tumour cells via EVs and play a role in altering the tumor microenvironment. Further research into the role of additional miRNAs that are selectively packaged into EVs is required to better understand cell-cell communication between the tumor and its microenvironment.

## MATERIALS AND METHODS

### Cell culture

All LAC cell lines and 293T cells were obtained from ATCC. LAC cell lines H1395, H1437, H2073, H2228 and H2347 were cultured in RPMI 1640 media supplemented with 10% fetal bovine serum (FBS). HMEC-1 cells were generously donated by the Aly Karson Lab, and HPL1D cells were donated by the Will Lockwood lab. Cell lines HMEC-1 and 293T were cultured in DMEM supplemented with 10% FBS. For EV collection, cells were grown in media supplemented with 1% depleted FBS (dFBS), which is FBS that has been depleted of EVs through overnight centrifugation at 110,000g and at 4°C. Cell counting was performed using a hemocytometer.

### EV collection

To collect EVs, “donor” LAC cells were grown in ten plates (15 cm), under normal conditions. Forty-eight hours prior to reaching confluence, media was replaced with RPMI 1640 containing 1% dFBS. Cells were allowed to secrete EVs into dFBS media for 48 hours, after which the conditioned media was collected. Our EV isolation protocol is based on a differential ultracentrifugation protocol described by Rani *et al*. [17]. Conditioned media was centrifuged at 300g for 10 min, then at 2,000g for 20 min and at 10,000g for 30 min at 4°C. Supernatant was collected at each stage of centrifugation and transferred to fresh 50 mL tubes for continued centrifugation. When completed, conditioned media was placed into open top ultracentrifuge tubes (Seton) and, using an ultracentrifuge set at 4°C, media was spun for 70 min at 110,000g. Supernatant was discarded, and the EV pellet was rinsed with PBS to reduce protein contamination before a second round of ultracentrifugation. The EV pellet was either processed to collect RNA (using a miRCURY RNA Isolation Kit [Exiqon]) or protein (using RIPA buffer), or re-suspended in 0.02 μm-filtered PBS to perform NanoSight analysis, or re-suspended in un-supplemented DMEM media to perform EV transfer assays.

### qRT-PCR

Cell line RNA and EV RNA was collected using miRCURY RNA Isolation Kit (Exiqon). Seven hundred and forty-two miRNAs were measured using microRNA Ready-to-Use PCR, Human panel I+II (Exiqon) on a Viia 7 Real-Time PCR System (Thermo Fisher). Cell line and EV profiles were normalized to global mean and fold-change analysis was used to compare matched cellular and EV miRNA profiles [36]. We subsequently validated EV miRNA candidates using a TaqMan Assay normalized to input and cel-miR-39-3p miRNA spike in to account for PCR efficiencies.

### Vectors

Over-expression (OE) cell lines were created using the FIV lenti-vectors HmiR0085-MR01 for miR-145, HmiR0084-MR01 for miR-143, or CmiR0001-MR01 (miR-Scramble) for a scramble sequence control (GeneCopoeia), vectors express both 3p and 5p miRNAs. Vectors were packaged using 293T cells and a Lenti-Pac FIV Expression Packaging Kit (GeneCopoeia). For knockdown assays, we used SMPD3 shRNA vectors TRCN0000048944, TRCN0000048945, TRCN000004896 and TRCN0000048947 (GE Dharmacon). Empty vector pLKO.1 was used as a control. The shRNA vectors were packaged using 293T cells and the plasmids VSVG and d8.91 using TransIT-LT1 transfection reagent (Mirus). Cells were infected over a 24 hour period, after which miRNA over-expression lines were selected using G418 for ten days, and shRNA lines were selected using puromycin for four days.

### Serum analysis

Serum samples were collected from patients undergoing LAC resection with curative intent (Table 2). All blood was collected in SST vacutainer tubes. For each patient, blood draws were taken from pulmonary venous effluent draining the tumor vascular bed, and from the systemic arterial blood of the same patient during surgery. Serum was isolated by centrifugation at 1500g for 15 min, and then frozen and stored at -80°C. RNA was isolated from 200 μl of serum using a Qiagen miRNeasy kit. MiRNAs were profiled for 742 miRNAs as described above. We removed miRNAs that are known to be affected by hemolysis by a fold change of greater than four [19]. Samples were normalized to miR-122-5p, which we have previously reported as an appropriate miRNA for normalizing patient matched samples, as it is not affected by hemolysis and shows little inter-patient variability [19].

**Table 2:** Patient demographics

Patient demographics		Patients
**Variable**	Patients	10
**Age (years)**		
	Mean	71.55555556
	Median	73
	Range	62-79
**Sex**		
	Females	5
	Male	4
	N/A	1
**Smoking history**		
	Current/former	7
	Never	1
	N/A	2
**Stage**		
	I	4
	II	2
	III	0
	IV	0
	NA	4

### EV incubation

Previously collected EVs were suspended in 100 μl DMEM media with no added supplements. The media was added to 50% confluent HMEC-1 cells in a 96 well plate and incubated for 24 hours, after which protein and RNA were collected.

### Tube formation assays

In triplicate for each treatment, 1.2×10^5^ HMEC-1 cells were added onto 24-well plates containing 200 μl of 10mg/ml Matrigel (Corning). EVs suspended in unsupplemented DMEM were incubated with the HMEC-1 cells for 16 hours. DIC images were taken using an Axiovert S1000 microscope under the 10X objective, with an image being taken at the center of each well. Tube length was quantified using ImageJ with the ‘Angiogenesis Analyzer’ plugin, and triplicates were averaged.

### Western blot

1:100 diluted phosphatase inhibitor cocktails I&II (Millipore Sigma) and 1:100 protease inhibitors (Thermo Fisher) were added to RIPA buffer to decrease sample degradation. Protein was quantified using a Pierce BCA kit and 10 μg of each sample was separated with a 12% SDS-PAGE gel and transferred onto PVDF membranes. Membranes were blocked in 5% BSA, 1X TBS and 0.1% Tween-20 for 1 hour. Membranes were incubated overnight at 4°C with primary antibodies diluted in blocking buffer. We used anti-CD63 (1:1000 dilution, EXOAB-Cd63A-1, System Biosciences), anti-TSG101 (1:1000 dilution, ab83, Abcam), anti-CAMK1D (1:800 dilution, #3365, Cell Signaling) and loading control anti-Histone H3 (1:4000, # 4499 Cell Signaling) followed by HRP conjugated anti-rabbit (1:2000, #7074, Cell Signaling). Detection was done using an Amersham ECL kit and hyperfilm (General Electric). Western images were quantified using the ‘gel analysis’ function in ImageJ (http://imagej.nih.gov).

## SUPPLEMENTARY MATERIALS FIGURES AND TABLES



## Abbreviations

LAC: lung adenocarcinoma
EVs: extracellular vesicles
NTA: nanoparticle tracking analysis
