# Supplementary material for: Selective secretion of microRNAs from lung cancer cells via extracellular vesicles promotes CAMK1D-mediated tube formation in endothelial cells
Source: Oncotarget. 2017 Aug 7;8(48):83913–24. doi: 10.18632/oncotarget.19996 (PMC5663564; doi:10.18632/oncotarget.19996)
Supplement: Supplementary file 1 [file oncotarget-08-83913-s001.pdf]

Selective secretion of microRNAs from lung cancer cells via extracellular vesicles promotes CAMK1D-mediated tube formation in endothelial cells

SUPPLEMENTARY MATERIALS

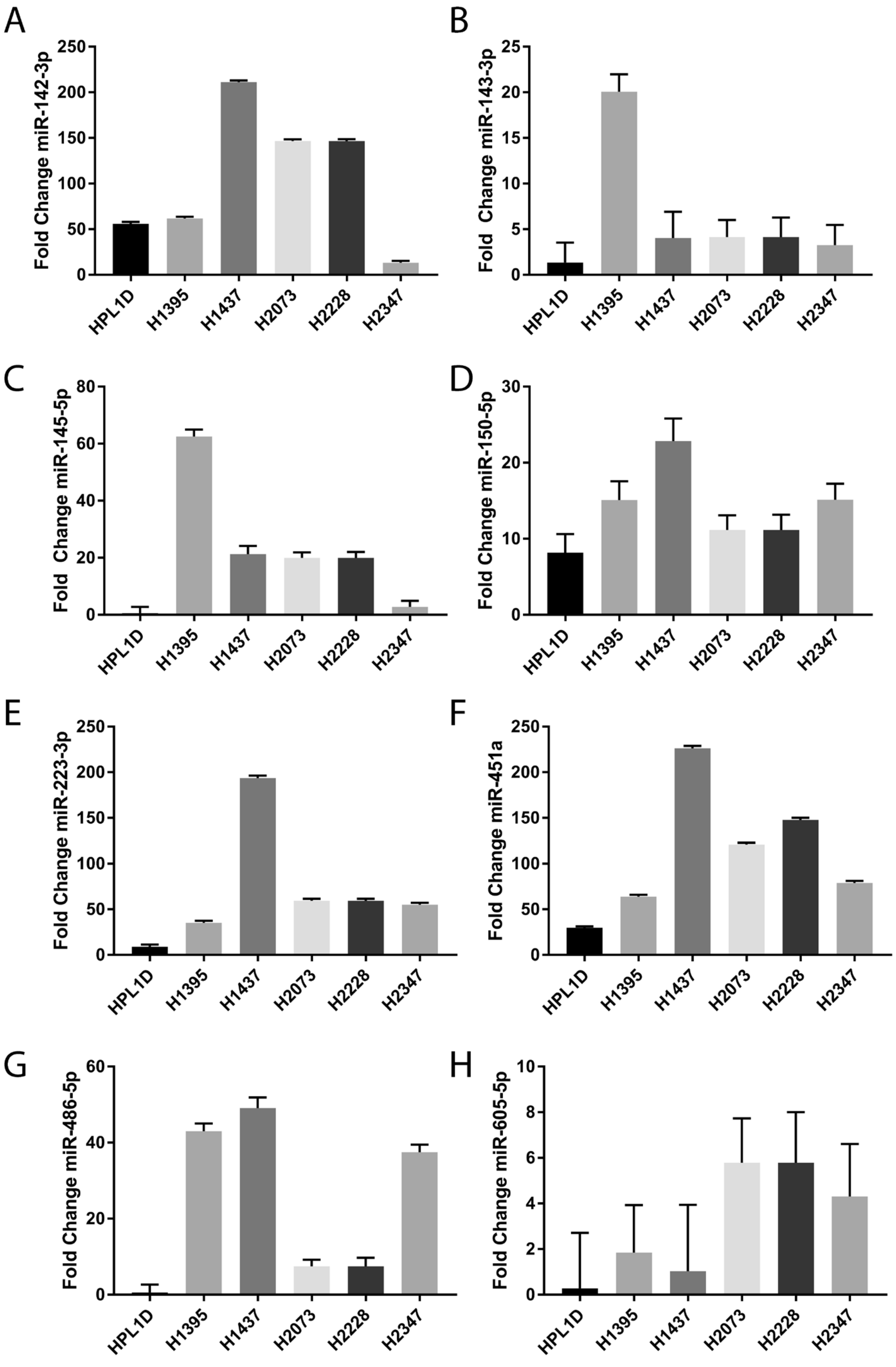

Supplementary Figure 1: Fold Change of EV candidate miRNAs in LAC cell lines H1395, H1437, H2073, H2228, H2347 and normal lung line HPL1D using Taqman qRT-PCR for miRNAs. **(A)** miR-142-3p **(B)** miR-143-3p **(C)** miR-145-5p **(D)** miR-150-5p **(E)** miR-223-3p **(F)** miR-451a **(G)** miR-486-5p **(H)** miR-605-5p.

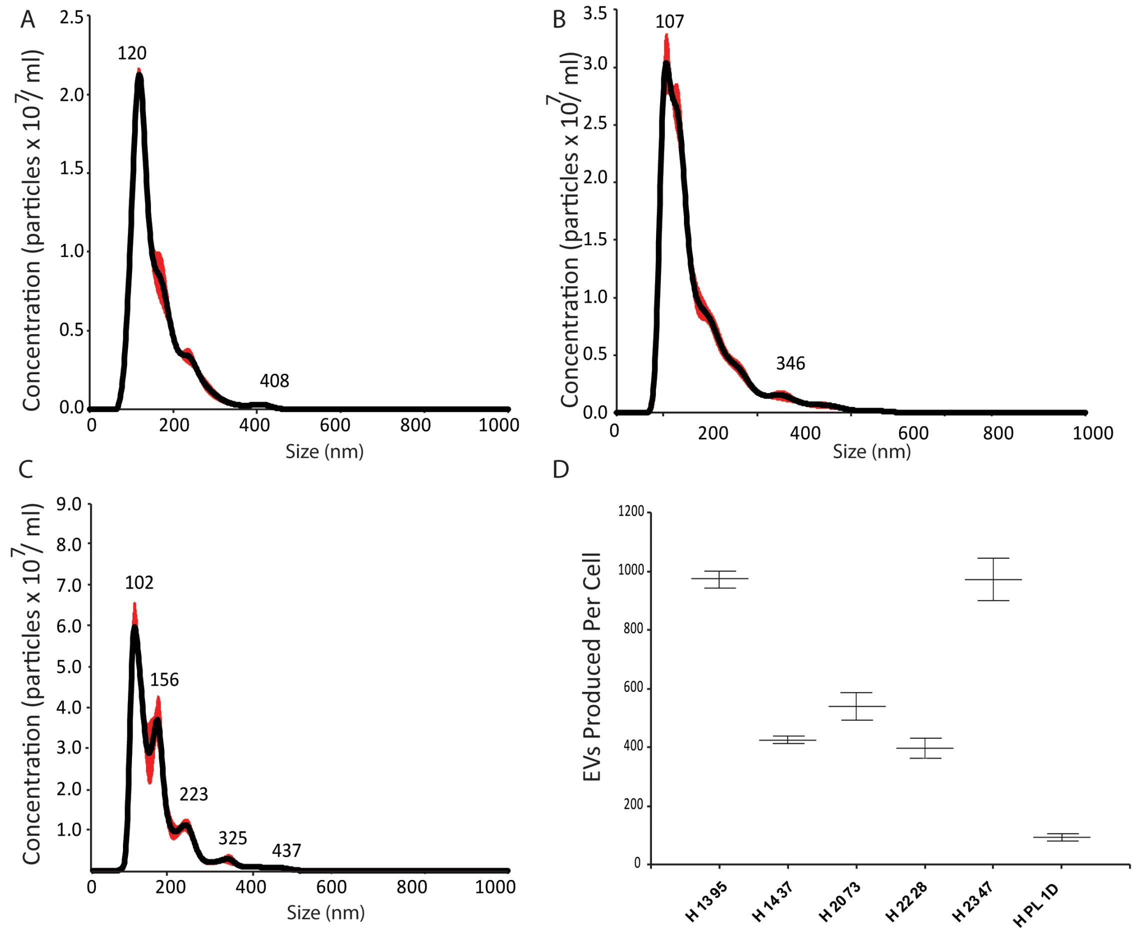

Supplementary Figure 2: Nanoparticle tracking analysis of cell line extracellular vesicle extractions using NanoSight. **(A)** H1395 cell line **(B)** H2228 cell lines **(C)** H2347 cell line **(D)** number of EVs per cell over 48 hours for all cell lines analyzed (H1395, H1437, H2073, H2228, H2347 and HPL1D).

Supplementary Table 1: Raw Ct value and global mean of candidate miRNAs from initial profiling of cell and extracellular vesicles (EV) from LAC cell lines

miRNAs	H1395		H1437		H2073		H2228		H2347	
	Cell Ct value	EV Ct value	Cell Ct value	EV Ct value	Cell Ct value	EV Ct value	Cell Ct value	EV Ct value	Cell Ct value	EV Ct value
miR-451a	ND	27.53	ND	27.18	ND	28.79	ND	26.80	ND	28.70
miR-142-3p	32.94	28.61	ND	28.77	ND	30.30	ND	29.05	34.73	30.58
miR-223-3p	ND	28.14	ND	29.56	ND	30.85	ND	29.83	ND	30.72
miR-605-5p	32.33	33.07	ND	30.83	ND	32.07	ND	29.87	ND	31.92
miR-150-5p	ND	29.31	33.77	31.27	ND	32.90	ND	31.14	ND	31.95
miR-144-3p	ND	29.95	ND	29.90	ND	31.78	ND	29.75	ND	31.99
miR-145-5p	ND	30.66	ND	31.72	ND	33.56	ND	34.00	33.81	31.83
miR-486-5p	34.97	30.33	ND	31.66	ND	32.83	ND	32.85	ND	33.26
miR-143-3p	ND	31.30	ND	31.21	ND	33.64	ND	32.14	32.69	32.46
**Global mean**	29.53	30.15	30.15	30.07	30.09	29.54	29.99	30.63	29.62	29.70

Supplementary Table 2: Mean fold change of miRNAs enriched in at least 5 of the 10 tumor draining samples

miRNA	Mean fold change	Up regulated by 4-fold in tumor draining serum (n=10)
**hsa-miR-375**	18.16	9
**hsa-miR-200c-3p**	36.86	9
**hsa-miR-125b-5p**	18.76	9
**hsa-miR-200b-3p**	20.97	8
**hsa-miR-141-3p**	64.67	8
**hsa-miR-10a-5p**	9.88	8
**hsa-miR-34a-5p**	12.95	7
**hsa-miR-204-5p**	9.30	7
**hsa-miR-99a-5p**	8.63	6
**hsa-miR-95-5p**	5.30	6
**hsa-miR-497-5p**	10.57	6
**hsa-miR-30a-3p**	8.69	6
**hsa-miR-145-5p**	9.14	6
**hsa-miR-143-3p**	13.78	6
**hsa-miR-99b-5p**	7.16	5
**hsa-miR-99a-3p**	8.73	5
**hsa-miR-365a-3p**	5.35	5
**hsa-miR-30a-5p**	11.71	5
**hsa-miR-199b-5p**	7.78	5
**hsa-miR-193b-3p**	7.88	5
**hsa-miR-18a-3p**	4.32	5
**hsa-miR-133a-5p**	6.01	5
